# 

**Published:** 1872-05

**Authors:** 


### THE AMERICAN JOURNAL OF DENTAL SCIENCE,

EDITED BY

F. J. S. GORGAS, M. D., D. D. S.

VOL. 6.—THIRD SERIES.

Surgeon Genl's Office LIBRARY. 39616 Wash[illegible]

BALTIMORE:
SNOWDEN & COWMAN.
LONDON:
TRUBNER & CO., 60 PATERNOSTER ROW.
1873.

3375

### INDEX TO VOLUME VI.---THIRD SERIES.

A Case of Adentulous Neuralgia, - - - - - 79
A Case of Cancrum Oris, - - - - - 182
A Curious Gum Varnish, - - - - - - 423
A New Method of Hardening Rubber, - - - 39
A New Method of Fitting Clasps to Teeth, - - - 80
A New Professorship, - - - - - 95
A New Appointment, - - - - - - 111
A Simple Remedy for Dandruff, - - - - 186
A Peculiar Case of Closure of the Jaws, - - - 279
A New Method of Arresting Epistaxis, - - - 280
A Pill for the Doctors, - - - - - 330
A Malformed Tooth, - - - - - 336
A Test for Pus, - - - - - - - 371
A Horrible Story from Paris, - - - - 376
A Simple Method of Arresting Epistaxis, - - - 474
An Interesting Operation, - - - - 41
Alabama Dental Association, - - - - 63
American Dental Association, - - - 104, 263
Arresting Hemorrhage after Extraction of Teeth, - - 192
Appeal in Behalf of the Wells' National Testimonial Fund, 223
American Academy of Dental Science, - - 224, 429
Antidote to Carbolic Acid, - - - - 236
Alcohol as a Medicine, - - - - - 281
Act to Regulate the Practice of Dentistry, - - 283
Arthur's Method of Treating Dental Caries, - - - 320
Anchylosis of Inferior Maxilla, - - - - 333
Arthur's Book, Dr. - - - - - - 350
Abnormal Secretion, - - - - - 394
Abstract of a Lecture on Pathology, - - - 401, 433
Artificial Eyes, - - - - - - 473
Advice to Students, - - - - - - 479
Advancement of Dental Science, - - - - 481
Anchylosis of Lower Jaw, - - - - - 521
An Ointment for Neuralgia, - - - - 523

Ancient Anæsthesia, - - - - - - 525
Althouse, M. D., M. R. C. P., Julius, - - - - 270
Annual Commencement of Baltimore College of Dental Surgery, 526
An Experiment and its Result, - - - - - 369
Aluminum Base, - - - - - - - 452
Baker, Dr. Elisha - - - - - - 48
Bogus Diplomas, - - - - - - 92, 480
Baltimore College of Dental Surgery, - - 334, 429, 526
Bond, M. D , Professor Thomas E. - - - 286, 384
Benecke, Dr. - - - - - - 470
Bleached Tincture of Iodine, - - - - 569
Bond, Dr. O. J. - - - - - - 13
Berry, D. D. S., A., - - - - - - - 40
Beers, L. D. S., W. George - - - - 81
Bramble, D. D., M. D., - - - - - - 172
BIBLIOGRAPHICAL.
Amnesic and Ataxic Aphasia and Hemiplegia, - 187
The Question of Quarantine, - - - - 187
The Farm and Fireside Journal, - - - 187
American Land and Law Adviser, - - - 187
The Pathology of the Teeth, - - - 426
Physician's Visiting List for 1873, - - - 427
Resection of the Maxillary Bones without External Incision, 427
Lecture on Water, - - - - - - 428
Ueber das Zahnen der Kinder, - - - - 428
The American Agriculturist, - - - 428
Woods Household Magazine, - - - - 428
A System of Oral Surgery of the Mouth, Jaws, and Associate Parts, - - - - - - 477
Herald of Health, - - - - - 477
Vick's Floral Guide, 1873, - - - - 478
Dental Caries and its Causes, - - - - 570
Cancrum Oris, - - - - - - 22
Careful Examinations, . . . . . . . . 46
Cummings' Patent, - - - - - - 47
Cutler, S. P., M. D., D. D. S., - - 3, 107, 159, 249
Chase, H. S., D. D. S., - - - - 30, 328
Cleaning and Bleaching Impression Wax, - - 95
Chase, E. C., D. D. S., - - - - 179, 232

Cement for Glass and Porcelain, - - - - 235
Case of Fracture and Subsequent Reunion of a Deciduous Incisor, 237
Cobb, S. J., D. D. S., - - - - - 256
Curtis, L., M. D., - - - - - - 273
Cancer from a Blow, - - - - - 281
Color Blindness, - - - - - - 282
Conservative Surgery of the Face, - - - 327
Chloral in Toothache, - - - - - 335
Cummings' Patent *versus* Dr. T. B. Gunning. - - 377
Cement for Attaching India Rubber to Wood and Metal - 425
Cough Mixture, - - - - - - 475
Carbolic Acid as a Local Anæsthetic, - - - 522
Cancrum Oris, Treatment of - - - - 525
Conservative Surgery of the Face, - - - 327
Death from Inhalation of Nitrous Oxide Gas, - - 13
Danger from the Vapor of Mercury, - - - 42
Death from Nitrous Oxide Gas, - - - - 55
Down, Dr. Langdon - - - - - 64
Diseases of the Antrum, - - - - - 147
Dandruff, A Remedy for - - - - - 186
Disinfectants, - - - - - - 184
Death from a Piece of Needle in the Heart, - - 332
Death from the Inhalation of Ether, - - - 332
Dental Surgery in the Royal College of Surgeons, - 335
Dental Quacks, - - - - - - 341
Davenport, J. H., M. D., - - - - 408
Dentistry Extraordinary, - - - - - 425
Dental Profession, - - - - - 431
Death from Ether, - - - - - 463, 566
Dunning, W. B., M. D., - - - - 463
Dean, M. S., D. D. S., - - - - 489, 553
Disinfection of Rooms, - - - - - 521
Diseases of the Gums, - - - - - 558
Death from Chloroform, - - - - - 560
Death from Nitrous Oxide, - - - - - 571
Enderman, H., Ph. D., - - - - - 165
Epulis, - - - - - - - 89
Exposed Nerves, - - - - - - 94
Epulis and Myeloid Tumors of the Jaw, - - - 112

Excision of the Upper Jaw—Recovery, - - - 176
Epithelioma Cured by Creasote, - - - 185, 376
Eames, W. H , D. D. S., - - - - 263
Eve, Paul, M. D., - - - - - - 276
Encephaloid Tumor of the Antrum, - - - 277
Excision of Upper Jaw, - - - - - 277
Extraction of Teeth, - - - - - 293
Excision of Inferior Maxilla, - - - - 365
External Use of Fuming Nitric Acid, - - - 425
Erratum, - - - - - - - 432
Electricity in the Treatment of Malignant Tumors, - 460
Effects of Tobacco, - - - - - - 467
Extirpation of the Parotid Gland, - - - 475
Ether Against Chloroform, - - - - - 568
Female Students, - - - - - 429
Fuller, A. H., D. D. S., - - - - - 80
Francis, C. E., D. D. S., - - - - 17
Foster, Joseph H., D. D. S., M. D., - - - - 96
Five Temporary Teeth at Forty, - - - - 569
Foil and Filling, - - - - - - 101
Floss Silk in Fang Filling, - - - - 104
Filling over Exposed Pulps, - - - - - 249
Fracture of Three Bicuspid Teeth Longitudinally by Contrecoup, 276
Formation of False Joint as a Cure for Anchylosis of Jaw, 522
Green, I. S. C., M. D., - - - - - 460
Gerhart, H., D. D. S., - - - - - 369
Garretson, James E., M. D., D. D. S., - - - 73
Gardner Appeal Case, - - - - - 113
Guilford. S. H., D. D. S , - - - - 215
Grant, H. M., D. D. S., M. D , - - - 309, 481
Georgia State Dental Society, - - - - 337
Howard, Dr. G. O. - - - - - 304, 341
Hypertrophy of the Maxilla, - - - - 45
Hampton, Dr. R. I. - - - - - - 337
Hollace, N. E., M. D., - - - - 1, 49, 442
Hunter, E. L., D. D. S., - - - 101, 261, 406
Hodgkin, James B., D. D. S., - - - 145, 241
Historical Notes on Poisoning, - - - - 225
Harris, E. N., D. D. S., - - - - 224

Human Ruminants, - - - - - - - 235
Hypertrophy of the Tongue, - - - - - 333
How to Administer Chloroform, - - - - - 381
Histological Processes in Divided Nerves, - - 470
Hemorrhagic Diathesis, - - - - - - 479
Heath, Christopher, F. R. C. S., - - - - 33
Itinerant Dentistry, - - - - - - - 9
Interesting Operation, - - - - - 41
Increase of Heart Disease, - - - - - - 92
Ingals, E. F., M. D., - - - - - 182
Iodized Cotton, - - - - - - - 236
Influence of Deficiency of Lime and Phosphoric Acid on the
Bones, - - - - - - 335
Influence of Mental over Man's Physical Forces, - - 351
In Memoriam—Professor Bond, - - - - - 384
Indigestion and its Management, - - - - - 415
Influences of Coffee and Cocoa on Nutrition, - - 520
Ingrowing Toe Nail, - - - - - - - 524
Is Woman Adapted to the Dental Profession? - - 542
Keesee, G. F., D. D. S., - - - - - - 385
Kilbourne, Dr. E. H. - - - - - 147
Large Crown Cavities with Walls Unbroken, - - 261
Liquid Nitrous Oxide Gas, - - - - - 191
Ligature of Common Carotid in Removal of a Superior Maxillary
Tumor, - - - - - - - 360
McCalla, John, D. D. S., - - - - - 293
Malformed Tooth, - - - - - - - 336
Meetings of Dental Associations, - - - - - 93
Microscopy of Minute Organisms, - - - - 107, 159
Mechanical Abrasion of the Teeth, - - - - 215
Malingering Detected by Faradization, and the Use of Nitrous
Oxide Gas, - - - - - - - 270
Mucous Membrane, Transplantation of - - - 282
McGuire, Hunter, M D., - - - - - - 277
Mixed Vapors for Anæsthesia, - - - - 424
Mercurial Inflammation of the Gums, - - - 498
Minot, Francis, M. D., - - - - - - 500
Mysterious Influences, - - - - - - 521
Morphine as a Local Anæsthetic, - - - - 332

Neuralgia, - - - - - 309, 442, 568
Neuralgia as Pertaining to the Dental Organs, - - 1
New Method of Hardening Rubber, - - - - 39
Nitrous Oxide Gas Experiments, - - - - 3
Nitrous Oxide Gas, Death from Use of - - - 55
Nitrous Oxide Gas, . . . . . . . . 145
Nitrous Oxide as an Anæsthetic, - - - - 165
Notes from Practice, - - - - - - 439
New Method of Arresting Epistaxis, - - - 474
Noel, Professor H. R., M. D., - - - 401, 433
New Method of Nickel Plating, - - - - 372
Neuralgia Cured by Shock, - - - - - 424
Newbrough, J. B., D. D. S., - - - - 55
On the Swallowing of Insoluble Foreign Bodies, - - 38
On the Effects of Diseased Meat as Food, - - 44
On the Relation of the Teeth and Mouth to Mental Development, 64
Ossified Pulp, - - - - - - 93
On the Extraction of Teeth, - - - - - 293
Oxygen as a Remedial Agent, - - - - 408
Obscure Cases of Dentalgia, - - - - - 443
Origin of Pus Corpuscles, - - - - 473
Old Rubber, - - - - - - - 476
On Measuring the Chest, - - - - - 475
On the Primary Dentition of Children, - - - 500
On Some of the Uses of Carbolic Acid, - - - 518
On Longevity, - - - - - - - 524
Osborn's Tongue Holder and Duct Compressor - - 576
OBITUARY.
Dr. Elisha Baker, - - - - - 48
Dr. Jos. H. Foster, - - - - - 96
Dr. E. T. Wilson, - - - - - 240
Professor Thomas E. Bond, A. M. M. D., - - 286
Pathology, Lecture on - - - - - 433
Pluggers, - - - - - - 30
Phosphor-Necrosis, - - - - - - 73
Purification of Carbolic Acid, - - - - 44
Prophylactic Measures for Decay of the Teeth, - - 47
Physiological Action of Putty, - - - - 185
Pharmaceutical and Medical Uses of Glycerine, - - 464

Proceedings of American Dental Association, - - - 263
Peculiar Case of Closure of the Jaws, - - - 279
Platinum Amalgam, - - - - - - 328
Plaster of Paris, - - - - - - - 356
Pivot Teeth, - - - - - - - 406
Pepsin in the Treatment of Exposed Pulps, - - - 528
Pathogenesis of Tobacco and Nicotine, Neurosis, &c., - 510
Pill for Neuralgia, - - - - - - - 568
Removal of Large Fibro-Cystic Tumor of Lower Jaw, - 33
Removing Salivary Calculus with Acids, - - - 45
Replantation, - - - - - - - - - 189
Removal of Inferior Maxilla for Malignant Epulis, - - 273
Rhinoplastic Operation, - - - - - - - - 362
Report of the Nitrous Oxide Committee, - - - - 417
Ready Mode of Preventing Infection, - - - - 425
Recurring Cancer Following Operation for Epithelioma, - 471
Southern Dental Association. - - - - 13, 111, 193
Second District Dental Society of New York, - - - 11
Severe Ptyalism from a Dental Operation without Amalgam, 40
Scales, J. H., D. D. S., - - - - - - - - 350
Sensitive Dentine, - - - - - - - - - 49
Sympathetic Effect of First Dentition, - - - - - 97
Southern Dental Association Meeting at Richmond, - - 239
System of Separating the Teeth, as Reviewed by O. A. Jarvis,
Dr. Arthur's, - - - - - - - - - 241
Sound Practice and Sound Philosophy, - - - 284, 489, 553
Stomatitis Materna, - - - - - - - - 289
Smooth Faced Plnggers, - - - - - - - - 304
Sick Headache, - - - - - - - - - - 331
Subcutaneous Injection of Morphia and Chloroform, - - 332
Suggestions, - - - - - - - - - - 349
Scurvy, - - - - - - - - - - 419
Saliva a Cure for Rheumatism, - - - - - - 475
St. Louis Dental Society, - - - - - - - - 563
Salivary Calculus. Cause of the Deposition of - - - 179
Salivary Calculus, - - - - - - - - - 189
The Cure of Stammering, - - - - - - - 566
The Medical Diploma Traffic, - - - - - - - 575
The Rubber Question, - - - - - - - - 576

Treatment of Sick Headache, - - - - - - - 515
The Cure of Stammering, - - - - - - - - 523
Treatment of Cancrum Oris, - - - - - - - 525
Treatment of Children's Teeth, - - - - - - 17
The Teeth and Mouths of Idiots, - - - - - - 27
Transformation of Medulla into Bone, - - - - - 91
The Abuse of Plastic Fillings, - - - - - - - 81
Tumor of the Upper Jaw, - - - - - - - - 172
The Blood in Syphilis, - - - - - - - - 186
Tumor of Inferior Maxillary, - - - - - - 188
Treatment of Cancrum Oris, - - - - - - - 236
Teething, - - - - - - - - - - - 256
Transplantation of Mucous Membrane, - - - - - 282
The Influence of Marriage Upon Health, - - - - 329
Training the Nervous System, - - - - - - - 375
Test for Creasote, - - - - - - - - 425
The Dental Profession, - - - - - - - - 431
The 33rd Annual Commencement of the Baltimore College of
Dental Surgery, - - - - - - - - 478
Thackston, W. W. H., M. D., D. D. S., - - - - - 529
Use of Tobacco, - - - - - - - - - - 476
Virginia State Dental Association, . . . 112, 336, 385
Voltaic Electricity and Amalgam Fillings, . . . . 232
Valedictory Address, . . . . . . . . . . 529
Wear and Repair of the Brain, . . . . . . . 567
Wilkerson, B. M., D. D. S., M. D,, . . . . . . . 104
Willson, Dr. O. . . . . . . . . . . . . 394
Westmoreland, Dr. . . . . . . . . . . . 439
Wedl's Pathology of the Teeth, . . . . . . . 573
Woman's True Rights, . . . . . . . . . . 574
Women as Doctors, . . . . . . . . . . . 576

### ARTICLE V.

### *Southern Dental Association.*

The fourth annual meeting of this body will be held in Richmond, Va., commencing on the last Tuesday in July, 1872, at 10 o'clock, A. M.

O. J. Bond, Recording Secretary.

---

### SELECTED ARTICLES.

---

### ARTICLE VI.

### *Death from the Inhalation of Nitrous Oxide Gas.*

### TO READERS AND CORRESPONDENTS.

Communications intended for publication, and exchange journals and papers, should be addressed to the EDITOR OF THE AMERICAN JOURNAL OF DENTAL SCIENCE, 259 N. Eutaw St., Baltimore, Md. All letters relating to the business of the journal, or containing cash remittances, to be directed to Messrs. SNOWDEN & COWMAN. Articles for publication should be received by the editor at least three weeks previous to the first day of the month on which the number in which it is designed for them to appear, is issued.

### CONTENTS:

### ORIGINAL COMMUNICATIONS.

Hypertrophy of the Maxilla.................................................................. 45

Removing Salivary Calculus with Acids................................................ 45

Careful Examinations........................................................................... 46

Prophylactic Measures for Decay of the Teeth...................................... 47

The Cumming's Patent......................................................................... 47

### OBITUARY.

Dr Elisha Baker................................................................................... 48

☞ The American Journal of Dental Science will contain fifty pages of reading matter in each number, making a volume of not less than

*Six Hundred* pages

EXCLUSIVE OF ADVERTISEMENTS.

THE

### AMERICAN JOURNAL OF DENTAL SCIENCE.

F. J. S. GORGAS, M. D., D. D. S., Editor.

The *Journal* is issued on the first of each month and contains not less than *fifty* pages of reading matter in each number, exclusive of advertisements, making a volume of *six hundred* pages per annum.

In each number will be found Original Communications, Correspondence, Selected Articles, Monthly Summary, Bibliographical Notices and Editorial Matter, and every effort will be exerted to render the *Journal* worthy of the patronage of the Dental profession.

A generous co-operation is respectfully solicited from the members of the profession; and as the *Journal* has now a printing office of its own which materially lessens the cost of its publication, it will be issued at the reduced subscription price of

**$2.50 Per Annum in Advance.**

Communications are respectfully solicited on all subjects pertaining to the practice of dentistry, as well as reports of cases occurring in dental practice.

### RATES OF ADVERTISING.

| | 1 MO. | 2 MO. | 3 MO. | 6 MO. | 12 MO. |
|---|---|---|---|---|---|
| 1 PAGE. | $15 00 | $22 50 | $30 00 | $50 00 | $100 00 |
| ½ " | 10 00 | 15 00 | 20 00 | 30 00 | 50 00 |
| ¼ " | 7 00 | 11 00 | 15 00 | 20 00 | 30 00 |
| ⅛ " | 5 00 | 8 00 | 11 00 | 15 00 | 20 00 |

All original communications designed for publication, works for review, new instruments for notice, exchanges, &c., should be directed to the editor, 259 N. Eutaw St., Baltimore, Md.

All advertisements and subscriptions should be addressed to

SNOWDEN & COWMAN,

*Publishers of the Am. Journal of Dental Science.*

86 W. FAYETTE ST. BET. CHARLES & LIBERTY STS.,

BALTIMORE, Md.

